# Survival analysis of PLWHA undergoing combined antiretroviral therapy: exploring long-term prognosis and influencing factors

**DOI:** 10.3389/fpubh.2024.1327264

**Published:** 2024-02-22

**Authors:** Jun-fan Pu, Jing Wu

**Affiliations:** ^1^Department of Infectious Disease, The People’s Hospital of Dazu District, Chongqing, China; ^2^The First Clinical College, Chongqing Medical University, Chongqing, China

**Keywords:** HIV/AIDS, survival, prognosis, antiretroviral therapy, cohort studies, LMIC

## Abstract

**Introduction:**

The survival time of human immunodeficiency virus (HIV)–infected individuals or patients with acquired immunodeficiency syndrome (AIDS) is influenced by multiple factors. Studying survival and influential factors after antiretroviral therapy (ART) contributes to improving treatment protocols, management strategies, and prognosis for people living with HIV/AIDS (PLWHA).

**Methods:**

This retrospective cohort study collected case data and follow-up records of PLWHA who received ART in Dazu District, Chongqing City, between 2007 and 2022. Cumulative survival rates were calculated using life tables. Survival curves were plotted using the Kaplan–Meier method. Uni-variable and multivariable Cox proportional hazards models analyzed factors influencing survival.

**Results:**

The study included 5,237 PLWHA receiving ART. Within the first year of ART initiation, 146 AIDS-related deaths occurred, accounting for 29.49% (146/495) of total deaths. Cumulative survival rates at 1, 5, 10, and 15 years were 0.97, 0.90, 0.85, and 0.79, respectively. During the observation period, male patients who received ART had a 1.89 times higher risk of death compared to females (aHR, 1.89; 95%; CI, 1.50–2.37). Patients aged ≥60 years had a 3.44-fold higher risk of death than those aged <30 years (aHR, 3.44; 95% CI, 1.22–9.67). Injection drug users (aHR, 4.95; 95% CI, 2.00–12.24) had a higher risk of death than those with heterosexual (aHR, 1.60; 95% CI, 0.69–3.72) and homosexual transmission. Patients with a baseline CD4+ T lymphocyte count <200 cells/μL (aHR, 8.02; 95% CI, 4.74–13.57) and between 200 and 349 cells/μL (aHR, 2.14; 95% CI, 1.26–3.64) had a higher risk of death than those with ≥350 cells/μL. Patients with ART initiation at WHO clinical stage IV had a 2.48-fold higher risk of death than those at stage I (aHR, 2.48; 95% CI, 1.17–5.23).

**Conclusion:**

The first year following ART initiation is critical in HIV/AIDS treatment, emphasizing the need for intensified follow-up and monitoring to facilitate successful immune system reconstruction. Older age, male sex, injection drug use, baseline CD4+ T lymphocyte count <200 cells/μL, and WHO clinical stage IV are associated with an increased risk of death. Tailored treatment and management strategies should be implemented for patient populations at higher risk of mortality and with a poorer prognosis.

## 1 Introduction

According to the estimates provided by the Joint United Nations Program on HIV/AIDS, the cumulative number of human immunodeficiency virus (HIV) infections since the start of the HIV/acquired immunodeficiency syndrome (AIDS) epidemic is estimated to be 85.6 million, with 40.4 million deaths attributed to AIDS-related illnesses (with a range of 32.9–51.3 million deaths). In the year 2022 alone, there were 1.3 million new HIV infections and 630,000 deaths (1). These figures highlight the substantial global impact of HIV and AIDS, underscoring the pressing need to address this persistent public health challenge.

China has emerged as a significant player in the global battle against HIV/AIDS. In China, there were 1.053 million PLWHA and 351,000 people died from HIV infection by 2020 (2). The southwestern region of China has been particularly hard-hit by the HIV/AIDS epidemic (3). Situated in the southwest, Chongqing is the largest municipality directly under the central government and plays a pivotal role in HIV prevention and control efforts nationwide. As of June 2023, Chongqing has reported a cumulative total of 59,073 individuals living with AIDS and 11,018 deaths, ranking among the cities most severely affected by the epidemic in China. Among its districts, Dazu District boasts the second-highest number of reported HIV infections.

In 2003, China implemented the “Four Frees and One Care” policy as a crucial measure in combating HIV/AIDS, vowing to supply PLWHA with complimentary antiretroviral drugs (4). The introduction of highly active antiretroviral therapy (HAART) has revolutionized the treatment and management of HIV/AIDS, leading to a significant improvement in patient prognosis. Accumulating evidence consistently demonstrates that early initiation of antiviral therapy offers a plethora of benefits, including prolonged survival, decreased mortality risk, restoration of impaired immune function to a normal state, prevention or reduction of opportunistic infections and select malignancies, and enhancement of overall quality of life (5–7).

The long-term trajectory of antiviral treatment for HIV/AIDS is influenced by a multitude of factors that impact the survival outcomes of affected individuals. Gaining insights into the posttreatment survival status and identifying the determinants of survival among PLWHA hold great significance for clinical decision-making, public health policies, prognosis and quality of life for these patients. Presently, there is a dearth of literature examining the survival status and its influencing factors in the context of PLWHA. In this context, we conducted a retrospective cohort study in Dazu District, Chongqing, an area burdened with a high prevalence of the epidemic. Our study aimed to elucidate the survival status and explore the associated factors among all PLWHA who received ART in the district between 2007 and 2022.

## 2 Materials and methods

### 2.1 Study population and data sources

The study population comprised individuals diagnosed with AIDS and HIV-infected individuals who commenced ART in Dazu District, Chongqing City, from January 2007 to December 2022. All PLWHA who received ART in accordance with the latest version of the “National Free Antiretroviral Therapy Manual for HIV/AIDS” (8) underwent standardized antiviral treatment. Data for the study were sourced from the Chinese Center for Disease Control and Prevention’s Comprehensive Information Management System for AIDS Prevention and Control, specifically downloading the medical records of adult PLWHA who underwent ART in Dazu District. These records included demographic information, laboratory test results, follow-up medication details, and death reports.

The inclusion criteria for the study participants were as follows: (1) laboratory-confirmed HIV infection by immunoblot assay; (2) case status (referring to the status of the patient case data being reviewed and confirmed by the Centers for Disease Control and Prevention) marked as “final review card”; (3) final review date on or before December 31, 2022; (4) current residence in “Dazu District”; (5) disease type classified as “confirmed case” or “clinically diagnosed case”; (6) age of ≥15 years; and (7) initiated ART between 2007 and 2022. The exclusion criteria were as follows: (1) case report card marked as “unreviewed card” or “deleted card,” (2) individual residing in a province or city outside the designated area, (3) uncertain laboratory testing results, (4) case classified as “suspected case,” (5) age of <15 years, and (6) severe baseline and follow-up information deficiencies. A total of 5,544 cases of PLWHA were initially enrolled between 2007 and 2022, and 307 cases met the exclusion criteria and were excluded, accounting for 5.54% of the total sample. 5,237 individuals ultimately included in this study.

### 2.2 Study design

A retrospective cohort study design was employed to collect data, the main components included the followings: (1) demographic and behavioral characteristics, such as sex, age, marital status, mode of infection, and source of cases. (2) disease information, such as the date of ART initiation, time from HIV diagnosis to ART initiation, cause and date of death, follow-up status (3)baseline CD4+ T lymphocyte count and World Health Organization (WHO) clinical staging at ART initiation.

The follow-up period began at the initiation of ART treatment for HIV/AIDS and ended on December 31, 2022. The primary outcome event was defined as AIDS-related death, including deaths resulting from opportunistic infections, AIDS-related tumors, AIDS-related specified diseases, and syndromes. The censored event was defined as an event in which a patient was dying from other causes (cardiovascular diseases, malignancies other than AIDS-related tumors, respiratory system diseases, endocrine and metabolic disorders, gastrointestinal diseases, other unrelated diseases, as well as deaths resulting from suicide, drug overdose, medication side effects, and injuries unrelated to diseases), was lost to follow-up, and had treatment cessation.

### 2.3 Statistical analyses

Data were organized using Microsoft Excel spreadsheets. Statistical analysis was performed using RStudio version 4.3.0 software. Descriptive statistics for continuous variables were reported as means ± standard deviations or medians (interquartile ranges), whereas categorical variables were presented as frequencies and percentages. The mortality, survival, and cumulative survival rates and standard errors of the cumulative survival rates were calculated using the life table methods. The Kaplan–Meier methods were used to plot the survival curves, and the log-rank tests were employed to compare the survival times. The relationship between risk factors and survival time was analyzed using the uni-variable and multivariable Cox proportional hazards models and the proportional hazards model and the results of the Cox regression have been checked. The Cox proportional hazards models were generated using a forward stepwise approach based on maximum likelihood estimation. The significance level for variable selection in the stepwise regression analysis was set at *p* < 0.05.

## 3 Results

### 3.1 Demographic and clinical characteristics

Up until the follow-up endpoint on December 31, 2022, a total of 5,237 PLWHA who underwent ART were included in this study, yielding a cumulative follow-up duration of 23,631.69 person-years. The mean duration of follow-up was 4.22 years. Among the cohort as shown in Table 1, 68.07% (3,565/5,237) were male, and 31.93% (1,672/5,237) were female, resulting in a male-to-female ratio of 2.1:1. The mean age of ART recipients was 60.85 ± 14.54 years. The age distribution exhibited that 2.60% (136/5,237) were aged 15–29 years, 10.96% (574/5,237) were aged 30–44 years, 32.29% (1,691/5,237) were aged 45–59 years, and 54.15% (2,836/5,237) were aged ≥60 years. Heterosexual transmission was found to be the predominant mode, accounting for 93.97% (4,921/5,237) of the cases, followed by homosexual transmission at 2.61% (137/5,237) and injection drug use at 2.14% (112/5,237).

**Table 1:** Demographic and clinical characteristics of PLWHA who received antiretroviral therapy (*N* = 5,237).

Characteristic		Number	%
Sex	Male	3,565	68.07
	Female	1,672	31.93
Age (year)	15–29	136	2.60
	30–44	574	10.96
	45–59	1,691	32.29
	≥60	2,836	54.15
Marital status	Unmarried	430	8.21
	Married/partnered	3,621	69.14
	Divorced/widowed	1,126	21.50
	Unknown	60	1.15
Source of cases	Medical institutions	4,448	84.93
	Healthcare facilities	789	15.07
Infection route	Injection drug use	112	2.14
	Heterosexual	4,921	93.97
	Homosexual	137	2.61
	Other	67	1.28
Baseline CD4 (cells/μL)	<200	2,184	41.70
	200–349	2,200	42.01
	≥350	853	16.29
Time interval between HIV/AIDS diagnosis and ART initiation (days)	<30	2,954	56.41
	30–179	970	18.52
	180–359	236	4.51
	≥360	1,077	20.56
WHO clinical staging at ART initiation	I	100	1.91
	II	2,786	53.20
	III	2,092	39.95
	IV	259	4.94

AIDS, acquired immunodeficiency syndrome; ART, antiretroviral therapy; HIV, human immunodeficiency virus; WHO, World Health Organization; Medical institutions, organizations such as hospitals, clinics, and medical centers at all levels that provide medical diagnosis, medication, surgical treatment, rehabilitation, and other services; Healthcare facilities, organizations such as disease control centers, health departments, and community health centers that focus on disease prevention, health education, and health testing function.

Upon initiating ART treatment, the mean baseline CD4+ T lymphocyte count was 231.4 ± 143.7 cells/μL. Of the patients, 41.70% (2,184/5,237) had a baseline CD4+ T cell count of <200 cells/μL, whereas 42.01% (2,200/5,237) had a count ranging from 200 to 349 cells/μL. Additionally, 16.29% (853/5,237) of the patients had a baseline CD4+ cell count of≥350 cells/μL. The time interval between HIV diagnosis and the initiation of ART treatment ranged from 1 to 5,515 days. Among the patients, 56.41% (2,954/5,237) commenced ART within 30 days of their diagnosis, while 18.52% (970/5,237) initiated treatment within 30–179 days of their diagnosis. A smaller proportion (4.51%, 236/5,237) started ART between 180 and 359 days, and 20.56% of PLWHA started ART treatment 360 days or more after their diagnosis.

### 3.2 Survival overview

In 2007, the first case of AIDS-related mortality was reported locally. By the conclusion of the follow-up period, a total of 495 individuals with HIV/AIDS had succumbed to AIDS-related diseases, resulting in an AIDS-related mortality rate of 9.45% (495/5,237).

Among the 5,237 patients, there were 146 deaths related to HIV/AIDS within 1 year of commencing ART, accounting for 29.49% (146/495) of the overall deaths. Over the initial 5-year period, 424 deaths occurred, representing 85.66% (424/495) of the total fatalities. The cumulative survival rates at 1, 5, 10, and 15 years were 0.97, 0.90, 0.85, and 0.79, respectively, as depicted in Table 2.

**Table 2:** Survival analysis life table for PLWHA who received antiretroviral therapy.

Interval start time (year)	Number entering interval	Number withdrawing during interval	Number exposed to risk	Number of terminal events	Proportion terminating, %	Proportion surviving, %	Cumulative proportion surviving at the end of interval, %	Standard error of the cumulative proportion surviving at end of interval
0~	5,237	453	5010.50	146	0.03	0.97	0.97	0.00
1~	4,638	512	4382.00	113	0.03	0.97	0.95	0.00
2~	4,013	618	3704.00	65	0.02	0.98	0.93	0.00
3~	3,330	544	3058.00	51	0.02	0.98	0.91	0.00
4~	2,735	462	2504.00	49	0.02	0.98	0.90	0.00
5~	2,224	681	1883.50	28	0.01	0.99	0.88	0.01
6~	1,515	376	1327.00	26	0.02	0.98	0.87	0.01
7~	1,113	311	957.50	8	0.01	0.99	0.86	0.01
8~	794	307	640.50	4	0.01	0.99	0.85	0.01
9~	483	271	347.50	2	0.01	0.99	0.85	0.01
10~	210	104	158.00	1	0.01	0.99	0.84	0.01
11~	105	59	75.50	0	0.00	1.00	0.84	0.01
12~	46	30	31.00	2	0.06	0.94	0.79	0.04
13~	14	8	10.00	0	0.00	1.00	0.79	0.04
14~	6	2	5.00	0	0.00	1.00	0.79	0.04
15~	4	4	2.00	0	0.00	1.00	0.79	0.04

**Figure 1:**
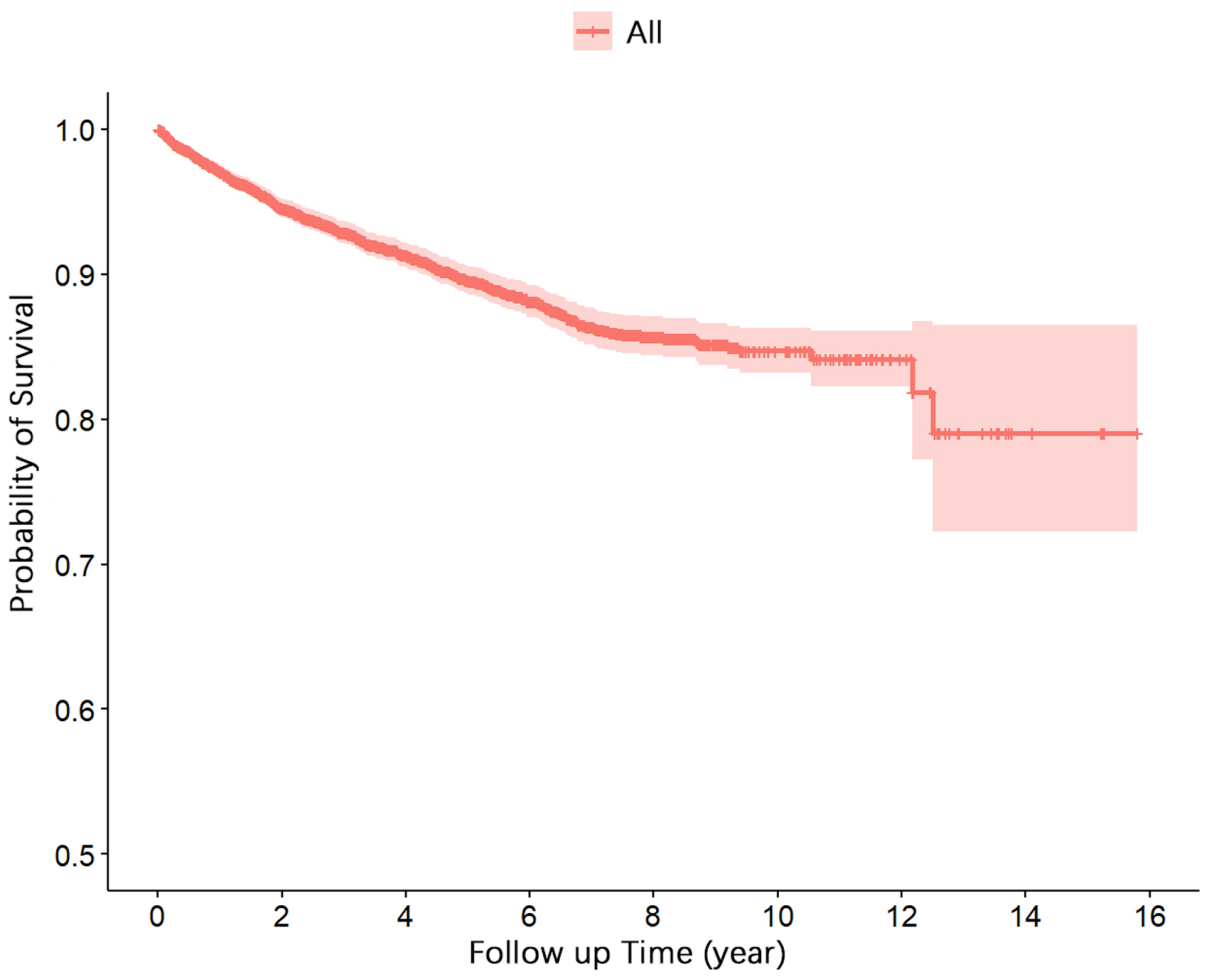
Survival curve for PLWHA receiving ART in Dazu District, Chongqing, from 2007 to 2022.

Figure 1 presents the survival function curve for the 5,237 cases of PLWHA. The curve demonstrates a rapid decline in the cumulative survival rates during the initial 5 years, followed by a gradual stabilization. From the 13th to the 15th year, the cumulative survival rate remained constant at 0.79.

**Figure 2:**
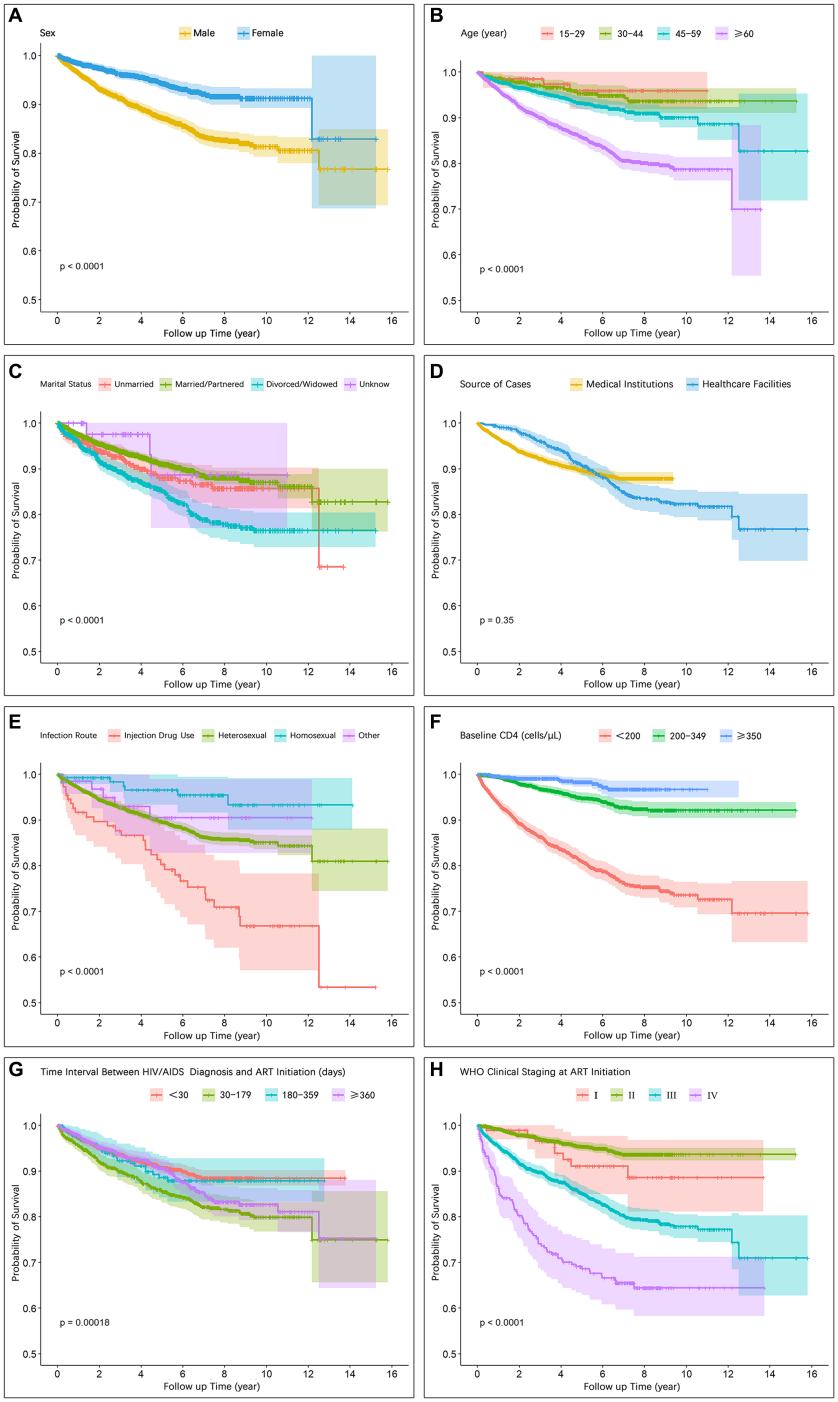
Survival curves showing intergroup differences with respect to **(A)** Sex, **(B)** Age, **(C)** Marital status, **(D)** Source of cases, **(E)** Infection route, **(F)** Baseline CD4, **(G)** Time interval between HIV/AIDS diagnosis and ART initiation, **(H)** WHO clinical staging at ART initiation.

Figure 2 illustrates the disparities in the survival function curves among the distinct groups. A comparison of the survival curves revealed that males exhibited significantly lower survival rates than females (*p* < 0.001; Figure 2A). Patients aged ≥60 years demonstrated significantly lower survival rates than the other age groups (*p* < 0.001; Figure 2B). Individuals infected through injection drug use and heterosexual transmission displayed significantly lower survival rates than those infected through homosexual transmission (*p* < 0.001; Figure 2E). Higher baseline CD4+ T cell counts were associated with increased survival rates (*p* < 0.001; Figure 2F). Patients classified in WHO clinical stages I/II exhibited significantly higher survival rates than those in stages III/IV (*p* < 0.001; Figure 2H).

### 3.3 Factors influencing survival

As shown in Table 3, in male patients who received ART, the risk of death was 1.89 times higher than that in female patients (adjusted hazard ratio [aHR], 1.89; 95% confidence interval [CI], 1.5–2.37). Furthermore, patients aged ≥60 years had a 3.44 times higher risk of death than those aged <30 years (aHR, 3.44; 95% CI, 1.22–9.67).

**Table 3:** Cox proportional hazards model analysis for PLWHA who received antiretroviral therapy (*N* = 5,237).

Variables	Patient, n	Death, n	HR (95% CI)	*p* value	aHR (95% CI)	*p* value
Sex						
Female	1,672	92	1.00		1.00	
Male	3,565	403	2.29 (1.82–2.87)	<0.001	1.89 (1.50–2.37)	<0.001
Age						
15–29	136	4	1.00		1.00	
30–44	574	25	1.28 (0.44–3.67)	0.648	0.86 (0.29–2.52)	0.784
45–59	1,691	104	2.00 (0.74–5.42)	0.174	1.39 (0.49–3.95)	0.535
≥60	2,836	362	4.51 (1.68–12.08)	0.003	3.44 (1.22–9.67)	0.019
Marital status						
Unmarried	430	43	1.00		1.00	
Married/partnered	3,621	291	0.78 (0.57–1.07)	0.128	0.44 (0.31–0.63)	<0.001
Divorced/widowed	1,126	158	1.45 (1.03–2.03)	0.031	0.76 (0.52–1.10)	0.149
Unknown	60	3	0.55 (0.17–1.78)	0.319	0.51 (0.16–1.67)	0.269
Source of cases						
Medical institutions	4,448	375	1.00		1.00	
Healthcare facilities	789	120	1.11 (0.89–1.37)	0.349	0.59 (0.45–0.76)	<0.001
Infection route						
Homosexual	137	6	1.00		1.00	
Heterosexual	4,921	454	2.84 (1.27–6.36)	0.011	1.60 (0.69–3.72)	0.276
Injection drug use	112	30	6.45 (2.68–15.49)	<0.001	4.95 (2.00–12.24)	0.001
Other	67	5	2.10 (0.64–6.90)	0.219	0.95 (0.28–3.22)	0.939
CD4+ count at baseline (cells/μL)						
≥350	853	16	1.00		1.00	
200–349	2,200	97	2.38 (1.40–4.04)	0.001	2.14 (1.26–3.64)	0.005
<200	2,184	382	9.94 (6.03–16.39)	<0.001	8.02 (4.74–13.57)	<0.001
Time interval between HIV/AIDS diagnosis and ART initiation (days)						
<30	2,954	217	1.00		1.00	
30–179	970	142	1.61 (1.30–2.00)	<0.001	1.72 (1.37–2.17)	<0.001
180–359	236	23	1.07 (0.69–1.64)	0.767	1.37 (0.89–2.13)	0.155
≥360	1,077	113	1.19 (0.95–1.50)	0.126	1.79 (1.39–2.29)	<0.001
WHO clinical staging at ART initiation						
I	100	8	1.00		1.00	
II	2,786	104	0.57 (0.28–1.17)	0.123	0.65 (0.31–1.36)	0.251
III	2,092	302	2.11 (1.04–4.26)	0.037	1.17 (0.57–2.40)	0.659
IV	259	81	4.36 (2.11–9.02)	<0.001	2.48 (1.17–5.23)	0.018

AIDS, acquired immunodeficiency syndrome; aHR, HR, adjusted hazard ratio; ART, antiretroviral therapy; HIV, human immunodeficiency virus; HR, hazard ratio; WHO, World Health Organization.

The mode of transmission serves as a crucial indicator influencing the HIV/AIDS mortality rates. Injection drug users (aHR, 4.95; 95% CI, 2.00–12.24) had a higher risk of death than those with heterosexual (aHR, 1.60; 95% CI, 0.69–3.72) and homosexual transmission (Table 3).

As shown in Table 3, a higher baseline CD4+ T cell count emerged as a risk factor for patient mortality. Patients with a baseline CD4+ T cell count of <200 cells/μL at the initiation of ART exhibited a 8.02 times higher risk of death than those with a CD4+ T cell count of ≥350 cells/μL (aHR, 8.02; 95% CI, 4.74–13.57). Patients with a baseline CD4+ T cell count ranging from 200 to 349 cells/μL had a 2.14 times higher risk of death than those with a CD4+ T cell count of <200 cells/μL (aHR, 2.14; 95% CI, 1.26–3.64).

As shown in Table 3, the time interval between HIV diagnosis and ART initiation ranging from 30 to 179 days had a 1.72 times higher risk of death than those with a time interval of <30 days (aHR, 1.72; 95% CI, 1.37–2.17), and the time interval between HIV diagnosis and ART initiation ≥360 days had a 1.79 times higher risk of death than those with a time interval of <30 days (aHR, 1.79; 95% CI, 1.39–2.29). Patients who started on ART while in WHO clinical stage IV had a 2.48 times higher risk of death than those in stage I (aHR, 2.48; 95% CI, 1.17–5.23).

### 3.4 Composition of AIDS-related death cases

**Figure 3:**
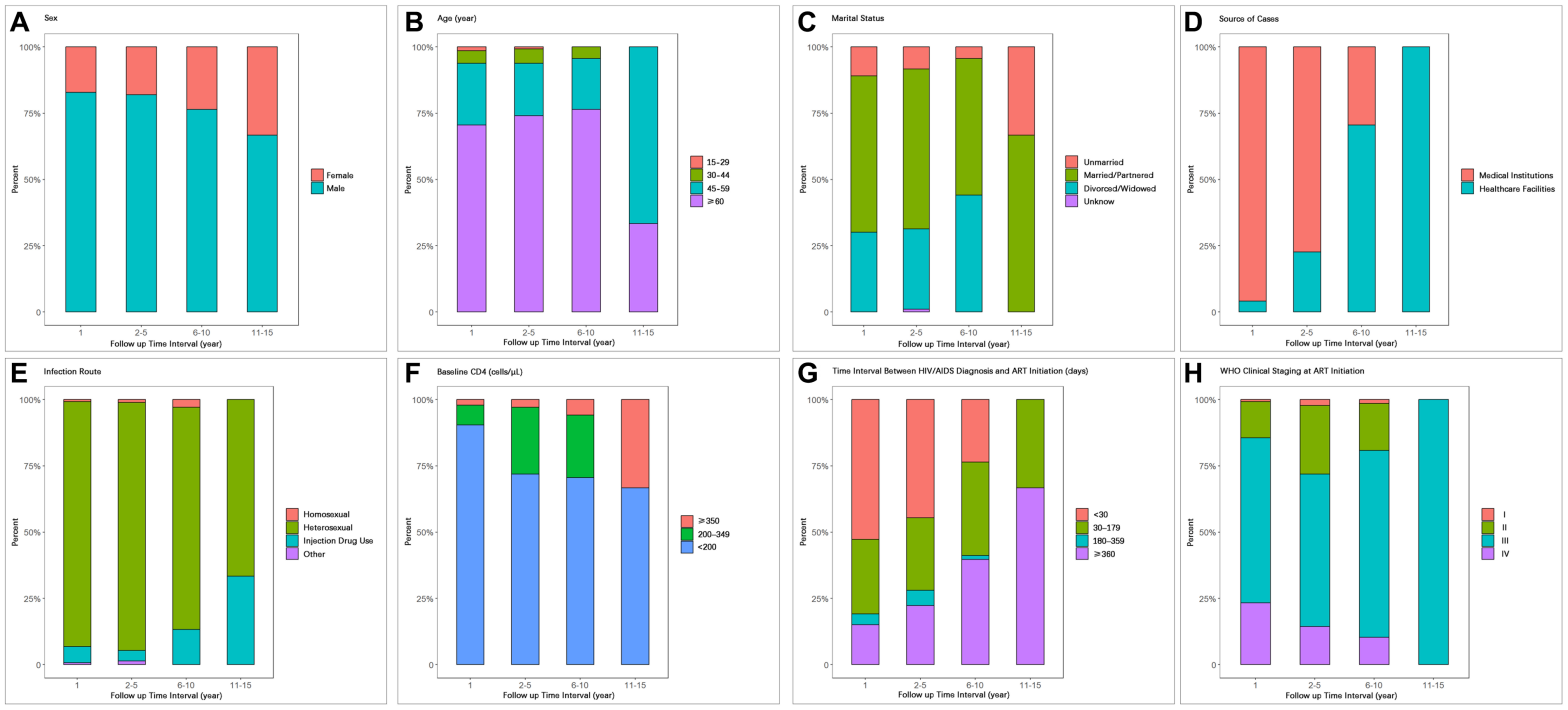
Composition of AIDS-related death cases during different time periods after ART initiation with respect to **(A)** Sex, **(B)** Age, **(C)** Marital status, **(D)** Source of cases, **(E)** Infection route, **(F)** Baseline CD4, **(G)** Time interval between HIV/AIDS diagnosis and ART initiation, **(H)** WHO clinical staging at ART initiation.

We conducted a statistical analysis of the composition characteristics of AIDS-related deaths among PLWHA in the first year, second to fifth year, sixth to tenth year, and eleventh to fifteenth year after initiating ART treatment, as shown in Table 4 and Figure 3. The number of AIDS-related deaths in the first year, second to fifth year, sixth to tenth year, and eleventh to fifteenth year were 146, 278, 68, and 3, respectively. Due to the relatively small number of AIDS-related deaths in the eleventh to fifteenth year, the data for this time period may not be representative. Therefore, we did not include the data for the eleventh to fifteenth year in the comparative analysis of different time periods. However, we still presented this data in the tables and figures.

With increasing follow-up time, the proportion of female deaths gradually increased (first year: 17.12%; second to fifth year: 17.99%; sixth to tenth year: 23.53%; Table 4; Figure 3A). The proportion of deaths among patients aged 60 and above also increased gradually (70.55, 74.10, 76.47%; Table 4; Figure 3B). The proportion of deaths among unmarried individuals decreased gradually (10.96, 8.27, 4.41%), while the proportion of deaths among divorced/widowed individuals increased gradually (30.14, 30.22, 44.12%; Table 4; Figure 3C).

**Table 4:** Composition of AIDS-related death cases during different time periods after ART initiation.

Characteristic		The 1st year, n (%)	2 ~ 5 year, n (%)	6 ~ 10 year, n (%)	11 ~ 15 year, n (%)	Total, n (%)
Sex	Male	121 (82.88)	228 (82.01)	52 (76.47)	2 (66.67)	403 (81.41)
	Female	25 (17.12)	50 (17.99)	16 (23.53)	1 (33.33)	92 (18.59)
Age (year)	15–29	2 (1.37)	2 (0.72)	0	0	4 (0.81)
	30–44	7 (4.79)	15 (5.40)	3 (4.41)	0	25 (5.05)
	45–59	34 (23.29)	55 (19.78)	13 (19.12)	2 (66.67)	104 (21.01)
	≥60	103 (70.55)	206 (74.10)	52 (76.47)	1 (33.33)	362 (73.13)
Marital status	Unmarried	16 (10.96)	23 (8.27)	3 (4.41)	1 (33.33)	43 (8.69)
	Married/partnered	86 (58.90)	168 (60.43)	35 (51.47)	2 (66.67)	291 (58.79)
	Divorced/widowed	44 (30.14)	84 (30.22)	30 (44.12)	0	158 (31.92)
	Unknown	0	3 (1.08)	0	0	3 (0.60)
Source of cases	Medical institutions	140 (95.89)	215 (77.34)	20 (29.41)	0	375 (75.76)
	Healthcare facilities	6 (4.11)	63 (22.66)	48 (70.59)	3 (100)	120 (24.24)
Infection route	Injection drug use	9 (6.17)	11 (3.95)	9 (13.24)	1 (33.33)	30 (6.06)
	Heterosexual	135 (92.47)	260 (93.53)	57 (83.82)	2 (66.67)	454 (91.72)
	Homosexual	1 (0.68)	3 (1.08)	2 (2.94)	0	6 (1.21)
	Other	1 (0.68)	4 (1.44)	0	0	5 (1.01)
Baseline CD4 (cells/μL)	<200	132 (90.41)	200 (71.94)	48 (70.59)	2 (66.67)	382 (77.17)
	200–349	11 (7.53)	70 (25.18)	16 (23.53)	0	97 (19.60)
	≥350	3 (2.06)	8 (2.88)	4 (5.88)	1 (33.33)	16 (3.23)
Time interval between HIV/AIDS diagnosis and ART initiation (days)	<30	77 (52.74)	124 (44.60)	16 (23.53)	0	217 (43.84)
	30–179	41 (28.08)	76 (27.34)	24 (35.30)	1 (33.33)	142 (28.69)
	180–359	6 (4.11)	16 (5.76)	1 (1.47)	0	23 (4.64)
	≥360	22 (15.07)	62 (22.30)	27 (39.70)	2 (66.67)	113 (22.83)
WHO clinical staging at ART initiation	I	1 (0.68)	6 (2.16)	1 (1.47)	0	8 (1.62)
	II	20 (13.70)	72 (25.90)	12 (17.65)	0	104 (21.01)
	III	91 (62.33)	160 (57.55)	48 (70.59)	3 (100)	302 (61.01)
	IV	34 (23.29)	40 (14.39)	7 (10.29)	0	81 (16.36)
Total		146 (29.49)	278 (56.16)	68 (13.74)	3 (0.61)	495

As the follow-up time increased, the proportion of deaths among PLWHA diagnosed in medical institutions showed a significant decrease (95.89, 77.34, 29.41%; Table 4; Figure 3D), while the proportion of deaths among PLWHA diagnosed through healthcare institutions showed a significant increase. In terms of transmission routes, the proportion of deaths among heterosexual transmission-infected individuals decreased from 92.47% in the first year to 83.82% in the sixth to tenth year, while the proportion of deaths among drug users increased significantly from 3.95% in the second to fifth year to 13.24% in the sixth to tenth year. The proportion of deaths among homosexual transmission-infected individuals showed a decreasing trend (Table 4; Figure 3E).

The proportion of deaths among PLWHA with a baseline CD4+ T-cell count of 350 and above increased year by year (2.06, 2.88, 5.88%), while the proportion of deaths among PLWHA with a baseline CD4+ T-cell count of less than 200 decreased year by year (90.41% gradually decreased to 70.59%). However, the proportion of deaths among patients with a CD4+ T-cell count of less than 200 remained significantly higher than those with a count of 200–349 and 350 and above across different time intervals (Table 4; Figure 3F).

Regarding the time interval between HIV/AIDS diagnosis and ART initiation, the proportion of deaths among patients diagnosed within 30 days showed a decreasing trend (52.74, 44.60, 23.53%), while the proportion of deaths among patients diagnosed 360 days and above showed an increasing trend (15.07, 22.30, 39.70%; Table 4; Figure 3G). With respect to WHO clinical staging at ART initiation, the proportion of deaths among patients in clinical stage III and IV gradually decreased (Table 4; Figure 3H).

## 4 Discussion

Based on the clinical population survey conducted in this study, a notable disparity was observed in the number of male patients (3,565) compared with that of female patients (1,672), which is consistent with the findings from other studies in Liangshan, Sichuan Province (9), Nanjing City (10), and Beijing city (11). The majority of individuals infected with HIV were found to be in the older adults age group, with 54.15% being over the age of 60 years. The mean age of patients who received ART treatment was 60.85 ± 14.54 years, which is higher than those in other studies (9–12). This difference may be attributed to the significant aging population structure in the local area.

Heterosexual transmission was identified as the predominant mode of HIV infection in this region, accounting for 93.97% of all transmission routes, whereas homosexual transmission only accounted for 2.61%, which is consistent with the findings from similar studies conducted in southwestern China, such as Liangshan, Sichuan Province (9), and Luzhou, Sichuan Province (13). However, in the eastern regions of China, including Nanjing City (10), Beijing City (11), and Tianjin City (14), the proportion of homosexual transmission exceeds that of heterosexual transmission. A report from China Medical University (15) emphasizes the substantial regional disparities in HIV transmission routes across China. In eastern and central provinces, homosexual transmission, particularly male-to-male sexual behavior, emerges as the primary mode of transmission, accounting for 62.4–77.88% of the newly reported cases. Conversely, in southwestern China, heterosexual transmission prevails as the main transmission route, accounting for 50–70% of the newly reported cases.

The results of the survival analysis demonstrated that the greatest number of deaths occurred within the first year of ART initiation, with 29.49% (146/495) of the total mortality attributed to AIDS-related illnesses. These findings are consistent with previous studies (9–11, 13, 14), emphasizing the critical and high-risk nature of the initial treatment year and the importance of enhanced follow-up and monitoring for newly treated patients, improved treatment adherence, and support for immune system reconstruction. The cumulative survival rates for patients at 1, 5, 10, and 15 years after ART initiation were 0.97, 0.90, 0.85, and 0.79 (Table 2), respectively. These rates are considered moderate compared with reports from economically disadvantaged remote regions such as Liangshan, Sichuan Province (9), and Gansu Province (16). However, they are lower than those observed in economically developed central cities in China, such as Nanjing (10), Beijing (11), and Tianjin (14). These findings suggest regional variations in the survival time and rates of PLWHA who received ART, with economically developed regions generally demonstrating longer survival times and higher survival rates. This discrepancy may be attributed to the availability of abundant medical resources and well-established healthcare systems in economically developed areas, enabling early detection of HIV infection and easier access to effective ART treatment. Moreover, the more comprehensive social support systems in economically developed regions can assist HIV-infected individuals in managing psychological challenges, overcoming stigma-related issues, actively seeking medical care, enhancing treatment adherence, and ultimately extending their survival time. Hence, in economically disadvantaged regions, there is a crucial demand to amplify resource allocation toward the prevention, treatment, and surveillance of HIV/AIDS, alongside augmenting the coverage of ART (17). A paramount imperative lies in advocating for HIV/AIDS education and knowledge dissemination to effectively tackle the issue of stigma, thereby facilitating patient access to comprehensive healthcare and social support networks, ultimately leading to enhancements in prognosis and overall survival outcomes.

The results of the Cox proportional hazards model analysis demonstrated a significant association between sex, age, transmission route, baseline CD4+ T lymphocyte count and WHO clinical stage and AIDS-related mortality among individuals living with HIV/AIDS (Table 3). Notably, this study revealed that during the observation period males faced a 1.89 times higher risk of death than females (aHR, 1.89; 95% CI, 1.5–2.37) and Cumulative survival rate was lower in males than in females (Table 3; Figure 2A), which is consistent with previous findings from both domestic and international studies (9–11, 18, 19). Consistent results were also reported by Shaik and colleagues (18) and Ingle and colleagues (19), further supporting the notion that males undergoing ART exhibit an increased mortality risk compared with females. Plausible explanations for this discrepancy include suboptimal adherence to ART treatment among male patients and a higher prevalence of unhealthy behaviors, such as smoking and alcohol consumption, leading to an increased vulnerability to mortality (20). Furthermore, it was observed that local male patients frequently engage in migrant work and business trips, which pose challenges in accessing timely and continuous medical interventions. Conversely, female patients tend to remain in their local areas to fulfill familial responsibilities, thus benefiting from more consistent access to healthcare services provided by community health institutions.

In terms of age, we identified an increased mortality risk associated with HIV/AIDS-related diseases among older individuals, with those aged ≥60 years having a 3.44 times increased risk compared with younger patients aged <30 years (aHR, 3.44; 95% CI, 1.22–9.67; Table 3). Notably, survival function analysis consistently revealed lower survival rates among the older age group across all time points (Figure 2B), indicating a poorer prognosis, which is in line with previous investigations (9, 10, 18, 21). The inferior prognosis observed in older patients stems from a combination of factors. Physiologically, age-related immunodeficiency and a higher prevalence of comorbidities impact their prognosis (22–24). Socially, older patients encounter obstacles when accessing healthcare services, such as challenges in seeking medical care independently, delayed diagnosis, limited treatment options, and inadequate social support systems. These challenges are particularly pronounced in resource-constrained middle-income regions and aging rural populations (23). Consequently, we assert that community and primary healthcare institutions should shoulder a greater responsibility in proactively providing improved medical assistance and social support for older patients.

Our findings significantly reveal notable disparities in HIV/AIDS-related mortality risks among individuals infected through different transmission routes (*p* < 0.001). Injection drug users exhibit a substantially increased risk of death compared with those infected through sexual transmission (aHR, 4.95; 95% CI, 2.00–12.24; Figure 2E; Table 3), which corroborates findings from collaborative studies conducted in Europe and North America (25). We attribute this discrepancy to the increased burden of health issues faced by individuals engaging in drug abuse and injecting behaviors, including pulmonary infections, tuberculosis, and liver disease (26). These complications and comorbidities further compromise patients’ immune systems, diminish the efficacy of ART, and increase the risk of HIV/AIDS-related mortality. Additionally, our analysis demonstrates that individuals infected through heterosexual transmission have a higher risk of death (aHR, 1.60; 95% CI, 0.69–3.72) than those infected through homosexual transmission, which is consistent with similar findings observed in other regions of China (9, 11). Homosexual population as a high-risk group for HIV/AIDS, display enhanced awareness of prevention and treatment, actively seek testing, and consequently achieve earlier diagnosis and treatment initiation. Hence, our results underscore the significance of transmission mode as a crucial indicator for HIV/AIDS prognosis. Clinicians should adopt more flexible approaches to diagnostic and treatment decision-making for patients infected through various transmission routes. For instance, individuals with a higher risk of death due to drug injection may require more frequent follow-up, treatment for complications, and management of comorbidities.

Multiple studies have consistently demonstrated a significant correlation between baseline CD4+ T cell count and HIV/AIDS-related mortality, revealing that individuals with lower baseline CD4+ T cell counts tend to have a higher risk of death and worse prognosis (27–32). Zhang and colleagues (27) identified a baseline CD4+ T cell count of <50 cells/μL as one of the most critical risk factors for HIV/AIDS-related mortality. Egger and colleagues (28) found that patients with higher baseline CD4+ T cell counts at ART initiation had a lower risk of disease progression or death. In our study, we also observed that patients with lower baseline CD4+ T cell counts at ART initiation had an increased risk of death. The survival function curve clearly illustrates that patients with baseline CD4+ T cell counts of ≥350 cells/μL and those falling within the range of 200–349 cells/μL had higher survival rates throughout the observation period than those with counts of <200 cells/μL (Figure 2F). Our analysis using the Cox proportional hazards model further revealed that patients with CD4+ T cell counts of <200 cells/μL exhibited a significantly higher risk of death (aHR, 8.02; 95% CI, 4.74–13.57) than those with counts of 200–349 (aHR, 2.14; 95% CI, 1.26–3.64) and ≥ 350 (Reference) cells/μL (Table 3). These findings emphasize that lower baseline CD4 counts independently contribute to the increased risk of patient mortality.

This study has some limitations. First, there were abnormalities and missing data in the follow-up information and clinical indicators of some patients. These patients were excluded from the analysis; although their number was minimal, it may still have the potential to affect the accuracy of the results. Second, existing literature suggests that factors such as HIV/AIDS viral load (33), CD4+/CD8+ T cell ratio (34), and adherence to ART treatment (35) may influence patients’ survival time and HIV/AIDS-related mortality. Regrettably, this study did not capture data on these factors. Third, we attempted to compare and analyze our study with global data. However, there is a lack of suitable literature for comparative analysis from other countries at present. We only referenced a few articles with large sample sizes and similar statistical methods from North America, Europe, and Asia to compare the research results. Last, this study excluded all-cause mortality cases of AIDS, as other disease causes may affect our accurate measurement of the impact of AIDS itself on patient survival. Perhaps in studies with larger sample sizes, it may be possible to consider a comparative analysis between all-cause mortality cases of AIDS and AIDS-related mortality cases to explore the competitive relationship between different causes of death.

## 5 Conclusion

This retrospective cohort study spanned a period of 16 years and encompassed nearly all HIV/AIDS cases in the region since the introduction of ART, ensuring a robust sample size. Within this study, we identified the initial year following ART initiation as a pivotal treatment phase, underscoring the significance of comprehensive follow-up and monitoring for recently treated patients to facilitate successful restoration of the immune system. Our findings revealed an increased risk of HIV/AIDS-related mortality and poorer prognosis in older individuals, males, individuals who acquired the infection through injection drug use, individuals with baseline CD4+ T lymphocyte counts of <200 cells/μL, and individuals in WHO clinical stage IV. In light of these results, it is imperative for clinicians to adopt proactive and efficacious treatment and management strategies when dealing with patient cohorts exhibiting an increased susceptibility to mortality.

## Data availability statement

The raw data supporting the conclusions of this article will be made available by the authors, without undue reservation.

## Ethics statement

The studies involving humans were approved by Ethics Commission of The People's Hospital of Dazu District. The studies were conducted in accordance with the local legislation and institutional requirements. The ethics committee/institutional review board waived the requirement of written informed consent for participation from the participants or the participants' legal guardians/next of kin because All identifiable information was anonymized. In compliance with national regulations and written informed consent was not required for this study. The animal study was approved by Ethics Commission of The People's Hospital of Dazu District. The study was conducted in accordance with the local legislation and institutional requirements.

## Author contributions

J-fP: Data curation, Formal analysis, Investigation, Methodology, Software, Writing – original draft, Writing – review & editing. JW: Data curation, Funding acquisition, Project administration, Supervision, Writing – review & editing.
